# Supplementary figures and images for: Maternal consumption of yoghurt activating the aryl hydrocarbon receptor increases group 3 innate lymphoid cells in murine offspring
Source: Microbiol Spectr. 2024 Oct 29;12(12):e00393-24. doi: 10.1128/spectrum.00393-24 (PMC11619593; doi:10.1128/spectrum.00393-24)

A

Luminescence signal  
(fold change vs Control yoghurt)

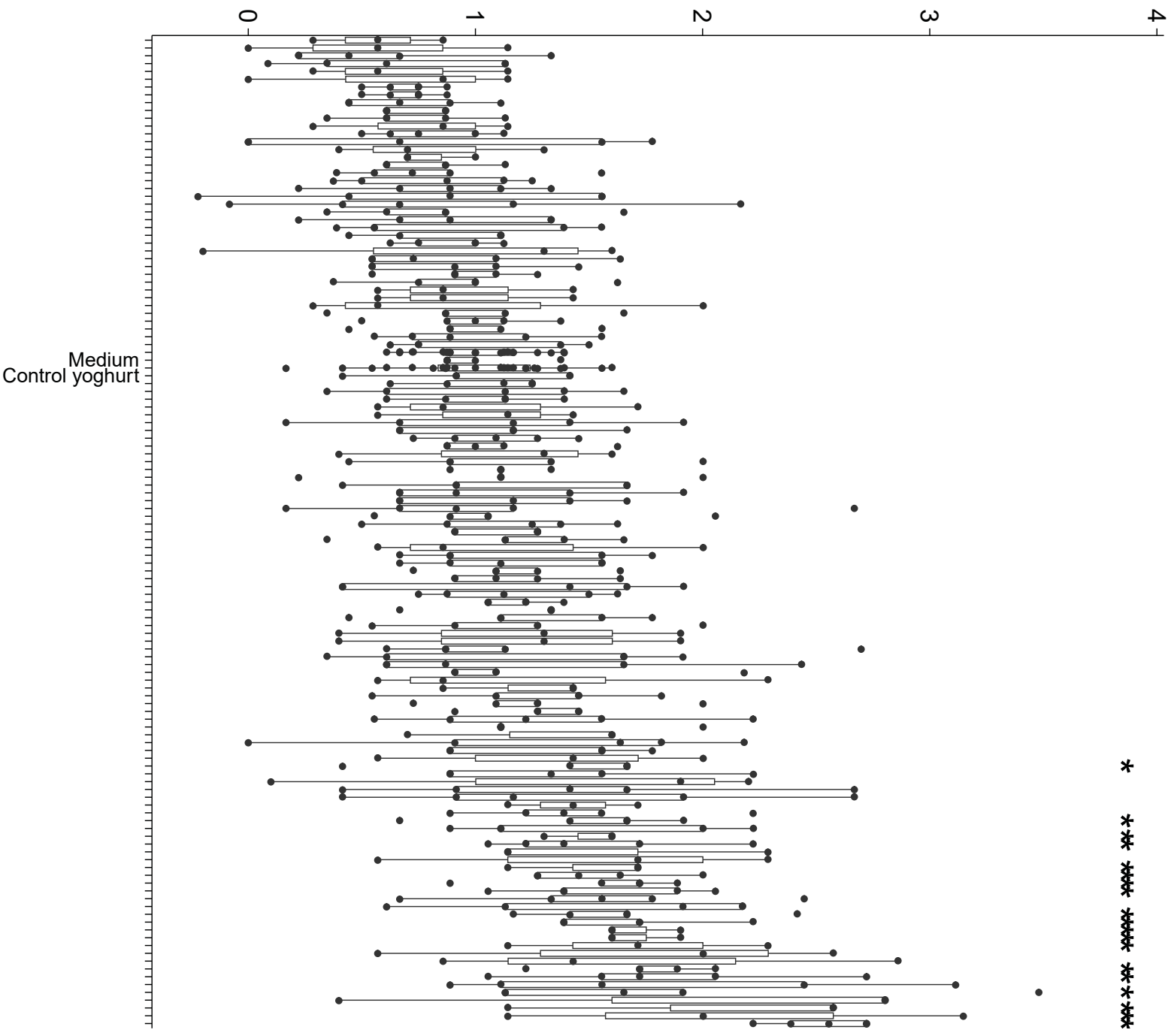

B

Luminescence signal (A.U.)

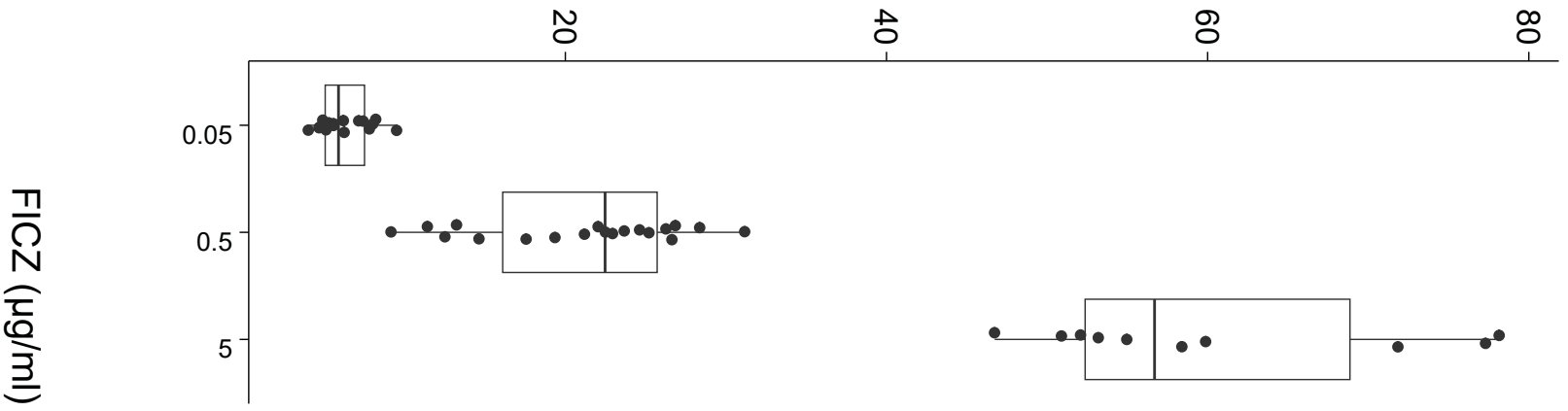

Supplement: Figure S1 — In vitro AhR activation assay of 125 test yoghurts as compared to the conventional control yoghurt. [file spectrum.00393-24-s0001.pdf]

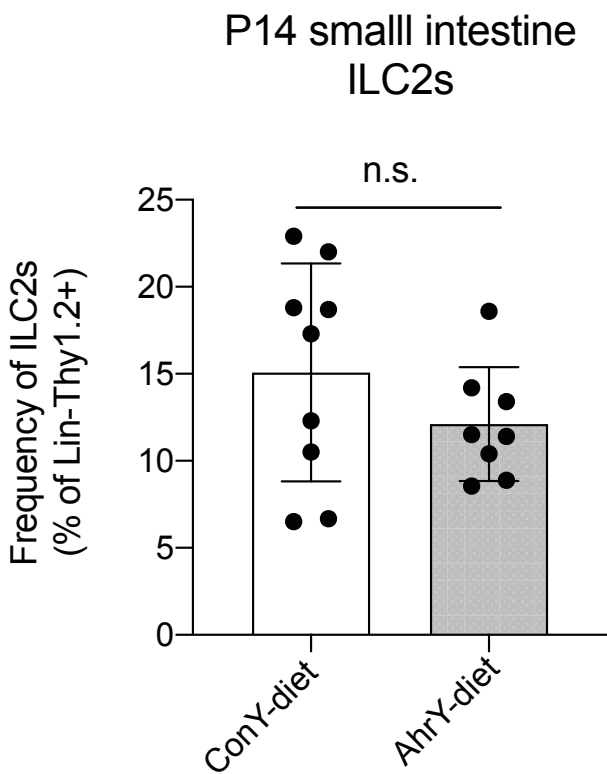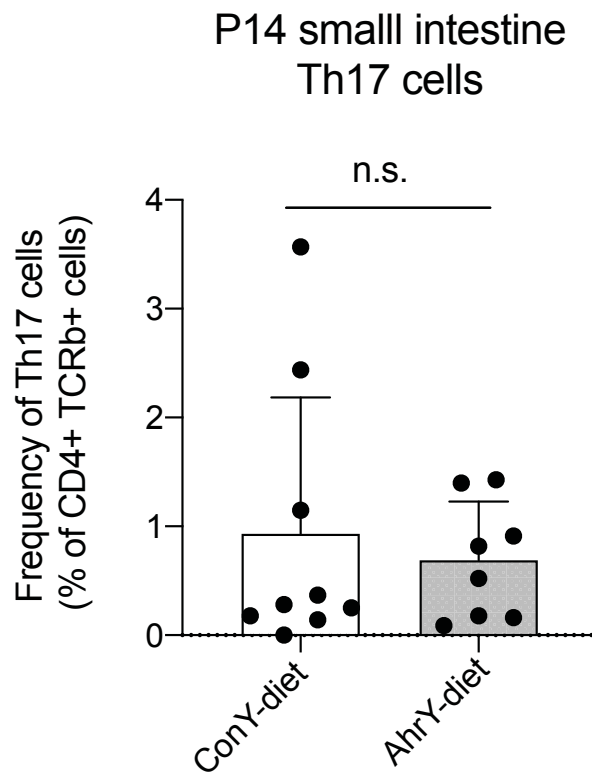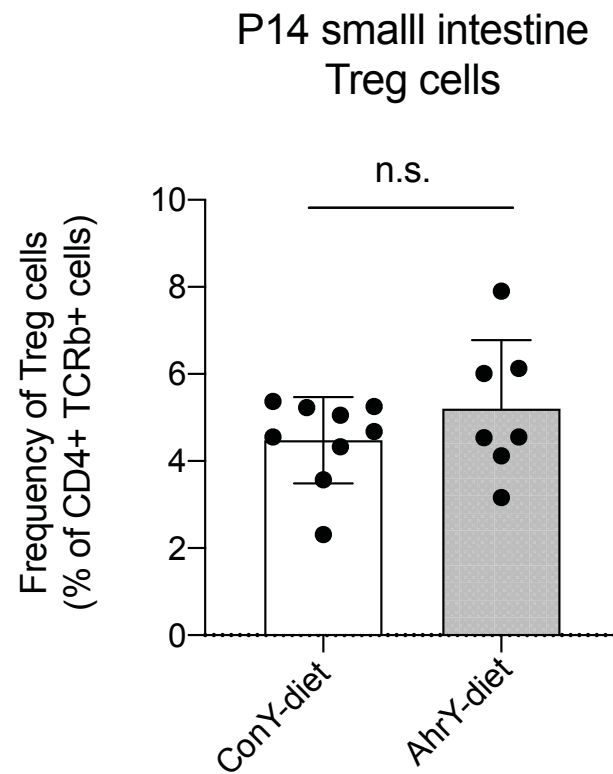

Supplement: Figure S2 — Feeding germ-free dams with the AhrY-diet. [file spectrum.00393-24-s0002.pdf]

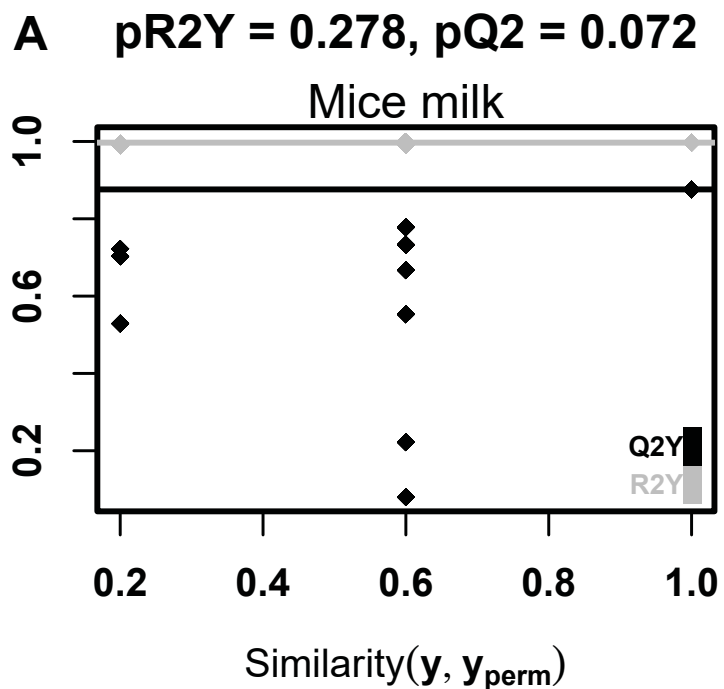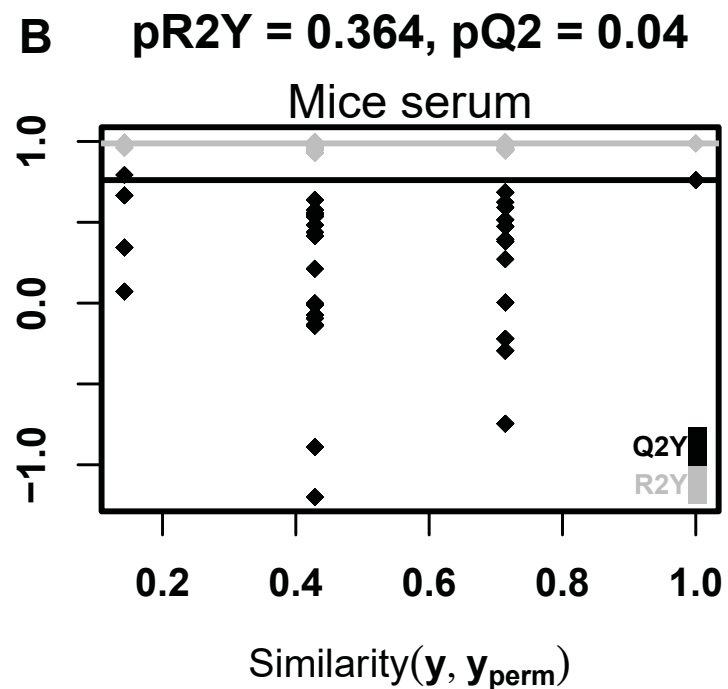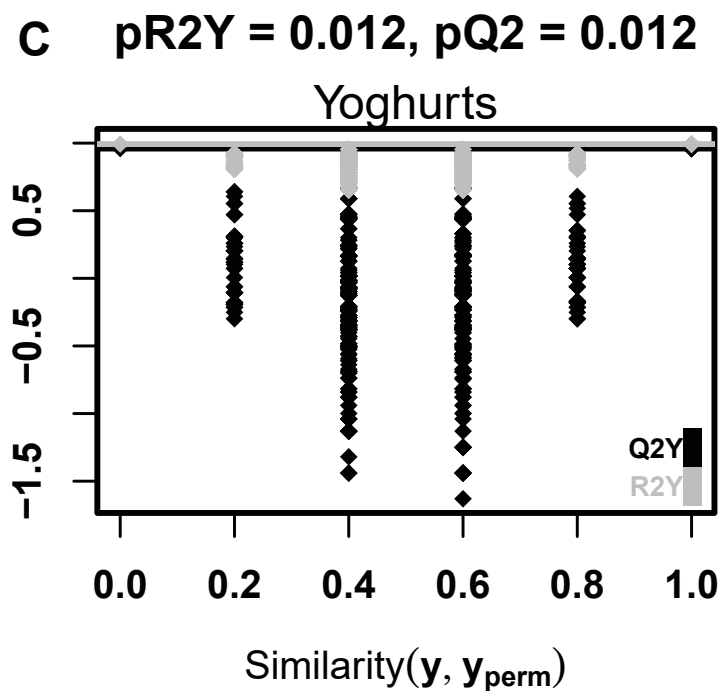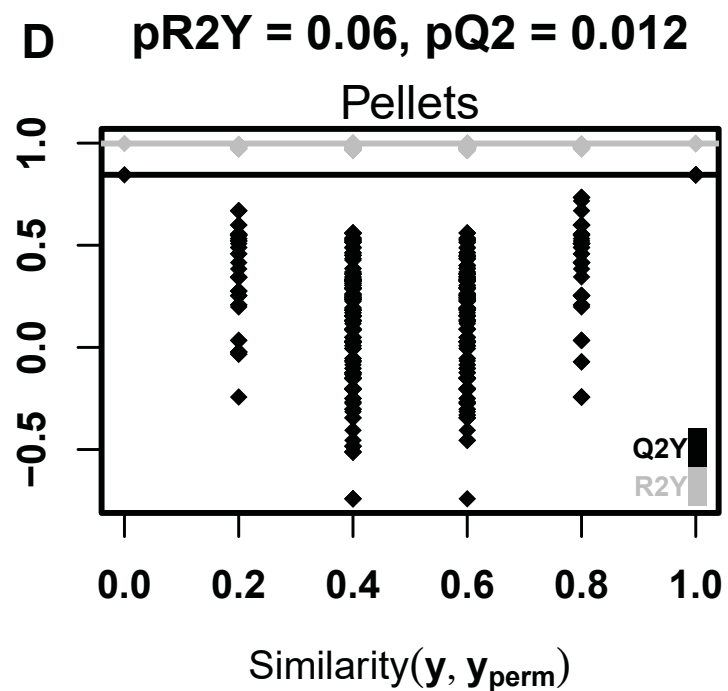

Supplement: Figure S3 — OPLS-DA permutation tests plots. [file spectrum.00393-24-s0003.pdf]
