# Supplementary material for: Maternal consumption of yoghurt activating the aryl hydrocarbon receptor increases group 3 innate lymphoid cells in murine offspring
Source: Microbiol Spectr. 2024 Oct 29;12(12):e00393-24. doi: 10.1128/spectrum.00393-24 (PMC11619593; doi:10.1128/spectrum.00393-24)
Supplement: Figure S4 — Functional analysis of compounds masses detected in murine milk. [file spectrum.00393-24-s0004.pdf]

A

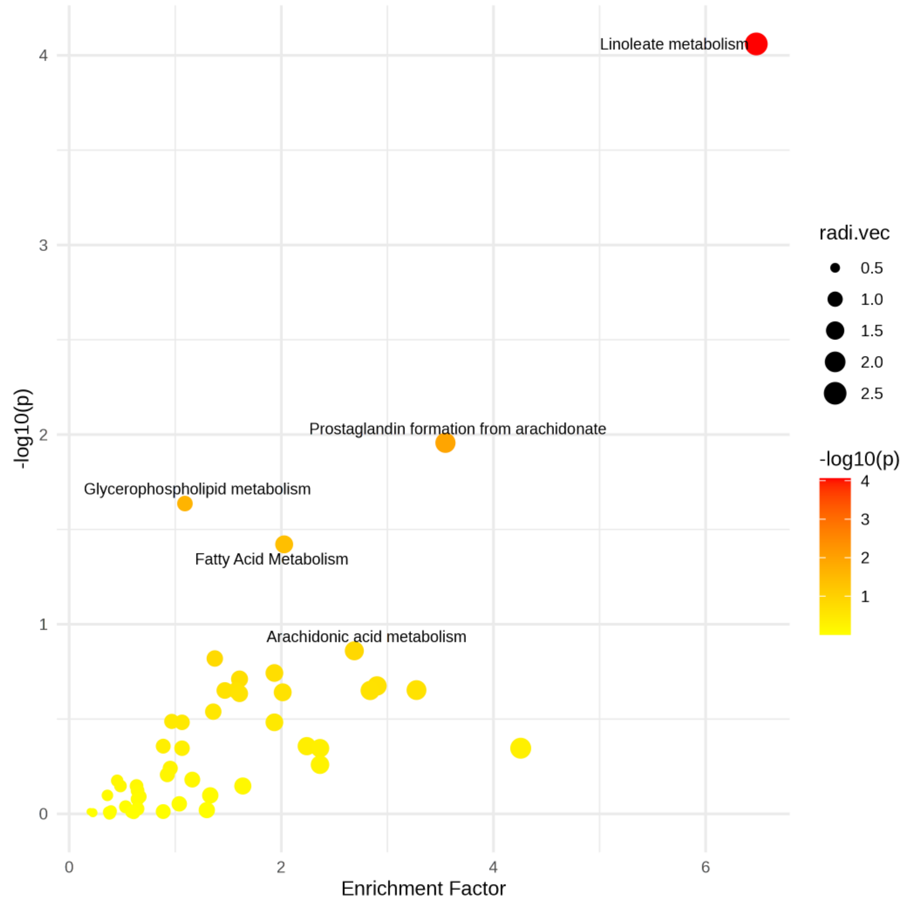

B

	Pathway total[a]	Hits.total[b]	Hits.sig[c]	Expected[d]	**P(Fisher)**[e]	P(Gamma)[f]
Linoleate metabolism	46	28	14	2.1613	**8.71E-05**	0.059524
Prostaglandin formation from arachidonate	78	38	13	3.6648	**0.011046**	0.060702
Glycerophospholipid metabolism	156	21	8	7.3296	**0.023103**	0.062488
Fatty Acid Metabolism	63	15	6	2.96	**0.037907**	0.06489

[a] Pathway total: The total number of empirical compounds in the pathway

[b] Hits.total: The total number of empirical compounds hits from the user's data

[c] Hits.sig: The total number of empirical compounds hits that are considered significant

[d] Expected: The expected number of empirical compounds hits in the pathway

[e] P(Fisher): The Fisher's Exact Test p-value for the pathay

[f] P(Gamma): P-values derived from Gamma distribution based on the permutation test for the pathway.
