# Supplementary material for: Maternal consumption of yoghurt activating the aryl hydrocarbon receptor increases group 3 innate lymphoid cells in murine offspring
Source: Microbiol Spectr. 2024 Oct 29;12(12):e00393-24. doi: 10.1128/spectrum.00393-24 (PMC11619593; doi:10.1128/spectrum.00393-24)
Supplement: Supplemental material — Legends for supplemental figures and tables. [file spectrum.00393-24-s0005.pdf]

LEGENDS OF SUPPLEMENTARY FILES

FIG S1 *In vitro* AhR activation assay of 125 test yoghurts as compared to the conventional control yoghurt. (A) 125 bacterial strains were tested to produce yoghurts that were then screened for their ability to activate AhR *in vitro* using the HepG2-AhR-Luc cell line. The figure presents their ability to activate AhR *in vitro* as compared to the control yoghurt (Wilcoxon test $p < 0.05$ as a threshold). (B) The AhR agonist FICZ was used at three different concentrations (0.05, 0.5 and 5 µg/ml) as a positive control.

FIG S2 Feeding germ-free dams with the AhrY-diet does not alter small intestinal ILC2s or T cell populations in the offspring. Time pregnant germ-free C57BL/6 dams were fed purified diets containing the AhR yoghurt (AhrY-diet) or control yoghurt (ConY-diet) starting on day 7 post conception until postnatal day 10. The offspring in each group was analyzed on postnatal day 14 by flow cytometry of the intestinal lamina propria. Relative frequency of small intestinal Gata-3+ ILC2s (A), Th17 cells (RORgt+ cells as frequency of CD4+TCRb+ cells) (B) and Treg cells (Foxp3+ cells as frequency of CD4+TCRb+ cells) (C) at postnatal day 14. Data represent mean ± SD, n=9 pups (ConY-diet), n=8 pups (AhrY-diet) from one experiment.

FIG S3 OPLS-DA permutation tests plots. OPLS-DA discriminating (A) milk from germ-free dams fed the AhR-diet and ConY-diet, (B) serum from germ-free dams fed the AhR-diet and ConY-diet, (C) the AhR-yoghurt from the control yoghurt, (D) the AhR-diet from

the ConY-diet. Permutation tests with 500 random permutations. The defined models were validated by higher original values of R2Y and Q2 than values obtained after permutations ($p \leq 0.06$). OPLS-DA, orthogonal projections to latent structures discriminant analysis; Q2Y, predictive ability parameter; R2Y, goodness-of-fit parameter.

FIG S4 Functional analysis of compounds masses (m/z) detected in murine milk after consumption of conventional yoghurt or AhR-yoghurt diet. (A) The enriched pathways predicted from mummichog algorithm (MetaboAnalyst 6.0 platform). (B) The four pathways showing significant enrichment (Fischer's Exact Test p-value < 0.05).

Table S1 List of tryptophan derivatives targeted by UHPLC-MS in the dairy products (milk, yogurt) as well as mice samples (milk, serum). The table indicates each compounds' supplier, precision of identification (mass and retention time errors) and, if detected, the Wilcoxon test p-value comparing their levels in control yoghurt versus AhR yoghurt or, in mice milk and serum, ConY-diet versus AhrY-diet.

Table S2 Macro- and micronutrient composition of the yoghurt-containing diets.
