# Supplementary material for: Maternal consumption of yoghurt activating the aryl hydrocarbon receptor increases group 3 innate lymphoid cells in murine offspring
Source: Microbiol Spectr. 2024 Oct 29;12(12):e00393-24. doi: 10.1128/spectrum.00393-24 (PMC11619593; doi:10.1128/spectrum.00393-24)
Supplement: Table S1 — List of tryptophan derivatives targeted by UHPLC-MS in the dairy products as well as mice samples. [file spectrum.00393-24-s0006.docx]

**Table S1** List of tryptophan derivatives targeted by UHPLC-MS in the dairy products (milk, yogurt) as well as mice samples (milk, serum). The table indicates each compounds’ supplier, precision of identification (mass and retention time errors) and, if detected, the Wilcoxon test p-value comparing their levels in control yoghurt versus AhR yoghurt or, in mice milk and serum, ConY-diet versus AhrY-diet.

| Compound name | Formula | HMDB ref | cas n° | Supplier | Mass error (ppm) | Pure standard retention time (min) | Peaks' retention time (% difference with standard) | | | p value (AhR yoghurt vs control yoghurt or AhRY-diet vs ConY-diet) | | |
| --- | --- | --- | --- | --- | --- | --- | --- | --- | --- | --- | --- | --- |
|  |  |  |  |  |  |  | Test yoghurts | Mice serum | Mice milk | Test yoghurts | Mice serum | Mice milk |
| 3-Hydroxyanthranilic acid | C7H7NO3 | HMDB01476 | 548-93-6 | Sigma - 148776 | < 5 | 4.1 | nd | nd | 2.4 | nd | nd | 0.800 |
| 3-Hydroxy-DL-kynurenine | C10H12N2O4 | HMDB0000732 | 484-78-6 | Sigma - H1771 | < 5 | 1.9 | nd | nd | nd | nd | nd | nd |
| 3-Indoleacetic acid | C10H9NO2 | HMDB0000197 | 87-51-4 | Sigma - I3750 | < 10 | 8.3 | nd | 0.1 | 0.0 | nd | 1.000 | 0.400 |
| 3-Indoleacetonitrile | C10H8N2 | HMDB06524 | 771-51-7 | Sigma - 129453 | < 3 | 10.3 | nd | nd | nd | nd | nd | nd |
| 3-Indoleacrylic acid | C11H9NO2 | HMDB00734 | 29953-71-7 | Sigma - I3807 | < 5 | 9.1 | 3.5 | nd | nd | 0.067 | nd | nd |
| 3-Indolepropionic acid | C11H11NO2 | HMDB0002302 | 830-96-6 | Sigma - 57400 | < 10 | 9.6 | nd | 0.0 | 0.0 | nd | 1.000 | 0.800 |
| 3-Methyl-2-oxindole | C9H9NO | HMDB0005785 | 700-06-1 | Sigma - 493937 | < 10 | 8.2 | nd | 0.7 | 2.1 | nd | 0.629 | 0.200 |
| 5-Hydroxyindole-3-acetic acid | C10H9NO3 | HMDB0000763 | 54-16-0 | Sigma - H8876 | < 3 | 5.2 | nd | 0.2 | nd | nd | 0.229 | nd |
| 5-Hydroxytryptophan | C11H12N2O3 | HMDB0000472 | 4350-09-8 | Sigma - 107751 | < 5 | 3.4 | 0.7 | nd | nd | **0.008** | nd | nd |
| 5-Methoxytryptamine | C11H14N2O | HMDB04095 | 608-07-1 | Sigma - 286583 | < 3 | 5.1 | nd | nd | nd | nd | nd | nd |
| 5-Methoxytryptophol | C11H13NO2 | HMDB0001896 | 712-09-4 | Sigma - M4126 | < 3 | 8.0 | nd | nd | nd | nd | nd | nd |
| Anthranilic acid | C7H7NO2 | HMDB0001392 | 150-13-0 | Sigma - A89855 | < 3 | 6.5 | 1.2 | nd | nd | **0.008** | nd | nd |
| Chorismic acid | C10H10O6 | HMDB12199 | 617-12-9 | Sigma - C1761 | < 3 | 4.4 | nd | nd | nd | nd | nd | nd |
| DL-Kynurenine | C10H12N2O3 | HMDB0000684 | 2922-83-0 | Sigma - 61250 | < 10 | 3.2 | nd | 0.3 | nd | nd | 0.229 | nd |
| Indole | C8H7N | HMDB0000738 | 120-72-9 | Sigma - I3408 | < 3 | 7.9 | nd | 0.3 | nd | nd | 0.400 | nd |
| Indole-3-acetamide | C10H10N2O | HMDB0029739 | 879-37-8 | Sigma - 286281 | < 10 | 6.4 | nd | nd | nd | nd | nd | nd |
| Indole-3-acetylglycine | C12H12N2O3 | HMDB0240661 | 13113-08-1 | Sigma - PH017265 | < 5 | 6.7 | 0.4 | 0.7 | 0.0 | 0.310 | 0.857 | 1.000 |
| Indole-3-butyric acid | C12H13NO2 | HMDB0002096 | 133-32-4 | Sigma - 57310 | < 3 | 10.5 | nd | nd | nd | nd | nd | nd |
| Indole-3-carbinol | C9H9NO | HMDB0005785 | 700-06-1 | Sigma - I7256 | < 10 | 6.7 | 0.1 | nd | 0.0 | 0.841 | nd | 0.200 |
| Indole-3-carboxaldehyde | C9H7NO | HMDB29737 | 487-89-8 | Sigma - 129445 | < 10 | 7.8 | nd | 0.1 | 0.0 | nd | 0.229 | 0.200 |
| Indole-3-lactic acid | C11H11NO3 | HMDB0000671 | 832-97-3 | Sigma - I5508 | < 1 | 7.3 | 0.0 | 0.3 | 0.0 | 0.690 | 0.857 | 1.000 |
| Indole-3-methyl acetate | C11H11NO2 | HMDB0002302 | 830-96-6 | Sigma - I9770 | < 5 | 11.2 | nd | nd | nd | nd | nd | nd |
| Indole-3-pyruvic acid | C11H9NO3 | HMDB0060484 | 392-12-1 | Sigma - I7017 | < 10 | 7.3 | 0.1 | nd | nd | **0.008** | nd | nd |
| Indoxyl | C8H7NO | HMDB0004094 | 480-93-3 | Santa Cruz Biotechnology - sc-490580 | nd | nd | nd | nd | nd | nd | nd | nd |
| Indoxyl Sulfate (potassium salt) | C8H6NO4S | HMDB0000682 | 487-94-5 | Sigma - I3875 | nd | nd | nd | nd | nd | nd | nd | nd |
| Kynurenic acid | C10H7NO3 | HMDB00715 | 492-27-3 | Sigma - K3375 | < 1 | 4.7 | 0.2 | 0.2 | 0.2 | **0.032** | 1.000 | 0.200 |
| l-tryptophan | C11H12N2O2 | HMDB0000929 | 73-22-3 | Sigma - 268683 | < 1 | 4.3 | 0.0 | 0.0 | 0.0 | **0.008** | 0.629 | 0.400 |
| Melatonine | C13H16N2O2 | HMDB01389 | 73-31-4 | Sigma - 814537 | < 5 | 8.0 | nd | nd | nd | nd | nd | nd |
| Nicotinamide | C6H6N2O | HMDB01406 | 98-92-0 | Sigma - 72340 | < 3 | 1.5 | 1.9 | 6.5 | 6.5 | **0.008** | 1.000 | 0.200 |
| Nicotinic acid | C6H5NO2 | HMDB01488 | 59-67-6 | Sigma - 72309 | < 3 | 1.4 | 9.2 | 7.9 | 0.0 | **0.032** | 0.857 | 0.800 |
| Picolinic acid | C6H5NO2 | HMDB0002243 | 98-98-6 | Sigma - P42800 | < 3 | 1.4 | 9.2 | 7.9 | 0.0 | **0.032** | 0.857 | 0.800 |
| Quinolinic acid | C7H5NO4 | HMDB00232 | 89-00-9 | Caymanchem - 14941 | nd | nd | nd | nd | nd | nd | nd | nd |
| Serotonin | C10H12N2O | HMDB0000259 | 50-67-9 | Sigma - 14927 | < 5 | 3.1 | nd | 2.6 | nd | nd | 0.629 | nd |
| Shikimic acid | C7H10O5 | HMDB0003070 | 138-59-0 | Sigma - S5375 | nd | nd | nd | nd | nd | nd | nd | nd |
| Skatole | C9H9N | HMDB0000466 | 83-34-1 | Sigma - W301912 | nd | nd | nd | nd | nd | nd | nd | nd |
| Tryptamine | C10H12N2 | HMDB0000303 | 61-54-1 | Sigma - 193747 | < 3 | 4.9 | nd | nd | nd | nd | nd | nd |
| Tryptophol | C10H11NO | HMDB0003447 | 526-55-6 | Sigma - T90301 | < 5 | 8.4 | 0.2 | nd | nd | **0.007** | nd | nd |
| Xanthurenic acid | C10H7NO4 | HMDB0000881 | 59-00-7 | Sigma - D120804 | < 3 | 4.3 | nd | nd | 0.0 | nd | nd | 0.200 |
